# Repeat hepatic resection *versus* percutaneous ablation for the treatment of recurrent hepatocellular carcinoma: meta-analysis

**DOI:** 10.1093/bjsopen/zrac036

**Published:** 2022-04-28

**Authors:** Bao-Hong Yuan, Yan-Kun Zhu, Xu-Ming Zou, Hao-Dong Zhou, Ru-Hong Li, Jian-Hong Zhong

**Affiliations:** 1 Department of General Surgery, Yan’An Hospital Affiliated to Kunming Medical University, The Key Laboratory of Tumour Immunological Prevention and Treatment of Yunnan Province, Kunming, China; 2 Department of Hepatobiliary Surgery, Guangxi Medical University Cancer Hospital, Nanning, China

## Abstract

**Background:**

The efficacy of repeat hepatic resection (rHR) in the treatment of recurrent hepatocellular carcinoma compared with radiofrequency or microwave ablation after resection of the primary tumour remains controversial. A systematic review and meta-analysis were performed to compare the safety and efficacy of these procedures.

**Methods:**

PubMed, Embase, Scopus, Cochrane Library, and China National Knowledge Infrastructure databases were systematically searched to identify related studies published before 10 October 2021. Overall and recurrence-free survival after different treatments were compared based on pooled hazard ratios with a random-effects model.

**Results:**

Two randomized clinical trials and 28 observational studies were included, involving 1961 and 2787 patients who underwent rHR and ablation respectively. Median perioperative mortality in both groups was zero but patients in the rHR group had higher median morbidity rates (17.0 per cent) than those in the ablation group (3.3 per cent). rHR achieved significantly longer recurrence-free survival than ablation (HR 0.79, 95 per cent c.i. 0.70 to 0.89, *P* < 0.001), while both groups had similar overall survival (HR 0.93, 95 per cent c.i. 0.83 to 1.04, *P* = 0.18).

**Conclusion:**

rHR and ablation based on radio- or microwaves are associated with similar overall survival in patients with recurrent hepatocellular carcinoma after resection of the primary tumour.

## Teaser

Repeat hepatic resection and ablation based on radio- or microwaves are associated with similar overall survival in patients with recurrent hepatocellular carcinoma.

Ablations associated with lower perioperative morbidity rates.

## Introduction

Hepatic resection and radiofrequency or microwave ablation are commonly used to treat patients with hepatocellular carcinoma (HCC) satisfying the Milan criteria (single nodule 5 cm or less, or up to three nodules less than 3 cm each, and no macrovascular invasion or distant metastasis)^1,2^. The 5-year recurrence rate is as high as 49–60 per cent among patients with early-stage HCC^3,4^. Given that HCC recurrence remains the leading cause of HCC-related deaths^5^, more effective treatment strategies are needed for recurrent HCC. Common therapies include repeat hepatic resection (rHR), radiofrequency or microwave ablation, liver transplantation, transarterial chemoembolization (TACE), radiotherapy, and administration of tyrosine kinase inhibitors. Although there are no definitive recommendations for the treatment of recurrent HCC^5–7^, rHR, ablation, and liver transplantation are considered the main curative approaches. The clinical application of liver transplantation is limited due to strict indications, lack of donors, and high treatment costs. In addition, meta-analyses on the safety and efficacy of rHR and ablation in patients with recurrent HCC within or beyond Milan criteria have provided conflicting conclusions^8–13^. In the present study, an updated systematic review with meta-analysis was performed to make recent comparisons of the safety and efficacy of rHR and microwave or radiofrequency ablation to treat recurrent HCC.

## Methods

### Study search

This meta-analysis was conducted according to the PRISMA Guidelines (*Supplementary Material*)^14^. A systematic search of PubMed, Embase, Scopus, Cochrane Library, and China National Knowledge Infrastructure databases was performed by two independent reviewers to retrieve articles published before 15 April 2021 using the following keywords: ‘hepatocellular carcinoma’ AND (‘recurrence’ OR ‘recurrent’) AND (‘repeat hepatectomy’ OR ‘repeat hepatic resection’, OR ‘re-hepatectomy’) AND ‘ablation’. The same search was repeated in October 2021 to identify studies published between 15 April and 10 October 2021. The search results were screened based on titles and abstracts, and appropriate articles were selected based on inclusion and exclusion criteria (see following section). The reference lists of relevant publications were also reviewed manually to identify additional potentially relevant articles.

### Inclusion and exclusion criteria

To be eligible for inclusion, studies had to involve patients with recurrent HCC after curative resection, followed by treatment with rHR, involving microwave ablation or radiofrequency ablation; compare the safety and/or efficacy of ablation and rHR for recurrent HCC; involve patients with recurrent HCC without macrovascular invasion or extrahepatic metastasis; and report one or more of the target outcomes of overall survival (OS), recurrence-free survival (RFS), or perioperative morbidity, or mortality. Eligible studies were included in the present meta-analysis even if patients received TACE or other treatments after rHR or ablation. In the case of studies with overlapping patient samples, only the largest study was included.

Exclusion criteria included studies comparing hepatectomy and ablation for primary or metastatic liver cancer; single-arm studies or studies where each treatment arm contained fewer than 10 patients; and studies in which patients received other therapies, such as TACE, radiotherapy, or tyrosine kinase inhibitors after HCC recurrence and before rHR or ablation.

### Quality assessment and data extraction

The eligibility of the included studies was assessed before data extraction. The quality of the randomized and non-randomized clinical trials (RCTs) was assessed, by use of the Cochrane Handbook for Systematic Evaluation of Interventions or the Newcastle–Ottawa Scale^15^. The following data were extracted independently by the two reviewers: first author name, sample size, age, sex, number and size of recurrent tumours, time to first recurrence, presence of liver cirrhosis, follow-up interval, perioperative morbidity, and mortality, as well as OS, RFS, and their hazard ratios (HRs). Disagreements were resolved by discussion or assessment by a third author.

### Primary and secondary outcomes

The primary outcome was OS, defined as the interval from rHR or ablation to treat recurrent HCC until death from any cause or until last follow-up. Secondary outcomes were perioperative mortality or morbidity and RFS, which was defined as the interval from rHR or ablation to treat recurrent HCC until HCC re-recurrence or death.

### Statistical analysis

Meta-analysis was performed with Review Manager version 5.3 (Cochrane Collaboration, Oxford, UK). Continuous data were reported as medians and quartiles, while differences between the two treatment groups were assessed for significance with the Mann–Whitney *U* test. Statistical heterogeneity was assessed with the *I*^2^ test. OS and RFS between the two groups were compared based on pooled HRs calculated with a random-effects model. Differences with *P* < 0.05 were considered statistically significant.

Whenever possible, unadjusted, or adjusted HRs were extracted from the original text of each study or estimated from Kaplan–Meier curves as described^16^. If both unadjusted and adjusted HRs were reported, the adjusted ratios were used. Median OS and RFS at 1, 3, and 5 years were estimated with bubble charts, where the size of each bubble represented the sample size of the given study^17^. The impact of individual studies on aggregate estimates was assessed through sensitivity analysis, in which the analysis was repeated after removing one study at a time. Funnel plots were also used to identify potential publication bias.

## Results

### Study selection

**Fig. 1:**
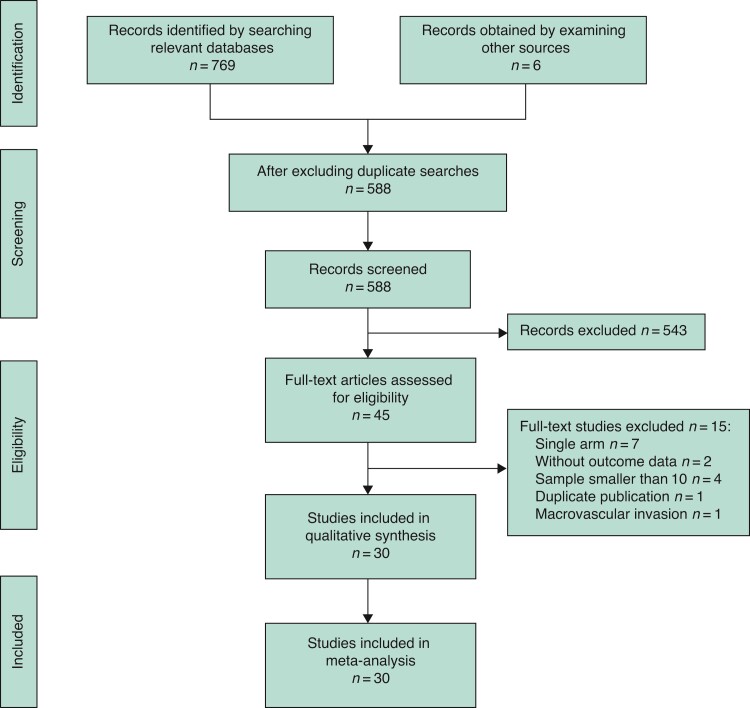
Flow diagram of selected studies for meta-analysis

After searching the indicated databases, a total of 767 studies were identified as potentially eligible, of which 185 were duplicates. Of the remaining 582 studies, 540 were excluded based on review of titles and abstracts, leaving 42 for full-text review. Of these 42 studies, 27 met the inclusion criteria and the rest were excluded due to duplicate publication or because they were single-arm studies, studies where each treatment arm contained fewer than 10 patients, studies with no outcome data, or studies on patients with recurrent HCC with macrovascular invasion. Two of the 27 selected studies were RCTs^18,19^ and 25 were observational comparisons^20–44^. Three additional studies were identified during the repeat literature search^45–47^. Overall, 30 studies were included in the meta-analysis (*Fig. 1*).

Based on the PRISMA Guidelines.

### Characteristics of included studies

One of the selected studies was conducted in Germany^23^ and the rest in China, Japan, Korea, Taiwan, Hong Kong, and Singapore^18–22,24–47^. Data were collected from 4748 patients, of whom 1961 were treated with rHR and 2787 with ablation (*Table 1*). Only one study involving 66 patients reported the use of microwave ablation^43^, whereas the remaining 26 applied radiofrequency ablation^18–42,44–47^. According to the Cochrane Handbook for Systematic Evaluation of Interventions, both RCTs were of high quality (*Table S1*). The Newcastle–Ottawa Scale score was above 5 for all non-RCTs, indicating acceptable quality (*Table S2*).

**Table 1:** Characteristics of included studies

Studies and country/region	Groups	Sample size	Age*, year	Solitary/multiple tumour	Recurrent tumour size, cm	Time to first recurrence, months* (%)	Cirrhosis, *n* (%)	Follow-up, months*
**Chan *et al***.^20^, **Hong Kong**	rHR	29	52	21/8	2.1 (0.8–5.5)	12.2	25 (86.2)	44.9
	Ablation	45	59	29/16	2.2 (0.8–6.0)	8.7	40 (88.9)	44.9
**Chen *et al.*^22^, China**	rHR	48	73.5	28/20	2.6 ± 1.135	-	41 (85.4)	36.9 (2–78)
	Ablation	57	73.7	30/27	2.5 ± 1.2	-	49 (86.0)	37.3 (2–78)
**Chen *et al.*^21^, China**	rHR	77	≤60 (67)	-	≤3 (39)	20	57 (74)	57 (2–168)
	Ablation	82	≤60 (61)	-	≤3 (77)	9	50 (61)	51 (4–111)
**Eisele *et al.*^23^, Germany**	rHR	27	60	16/11	4.0 ± 2.3	39	10 (37)	-
	Ablation	27	68	15/12	2.8 ± 1.1	21	22 (81.5)	-
**Feng *et al.*^24^, China**	rHR	99	56.0	75/24	3.0 (2.5–4.0)	>1 year (79)	60 (60.6)	-
	Ablation	191	57.9	121/70	2.2 (1.5–3.0)	>1 year (106)	126 (66)	-
**Hirokawa *et al.*^25^, Japan**	rHR	10	69	7/3	1.9 ± 0.7	22.8	3 (30)	-
	Ablation	21	67	16/5	1.7 ± 0.6	7.6	8 (38)	-
**Ho *et al.*^26^, Taiwan**	rHR	54	56.3	-	2.9 ± 1.8	23.9	26 (48.1)	32 (0–79)
	Ablation	50	61.0	-	2.3 ± 1.9	20.0	28 (56.0)	27 (0–96)
**Huang *et al.*^27^, China**	rHR	66	50.5	66/0	2.9 ± 1.1	17.1	57 (86.3)	-
	Ablation	46	54.1	46/0	2.6 ± 0.9	14.1	39 (84.8)	-
**Kawano *et al.*^28^, Japan**	rHR	13	-	-	-	-	-	-
	Ablation	33	-	-	-	-	-	-
**Kim *et al.*^29^, Korea**	rHR	45	53	45/0	2.0 (0.7–4.6)	22	-	64 (4–113)
	Ablation	171	56	170/1	1.4 (0.2–4.8)	18	-	60 (6–115)
**Liang *et al.*^30^, China**	rHR	44	48.8	34/10	≤3 (26)	-	-	33.5 ± 24.1
	Ablation	66	54.6	48/18	≤3 (44)	-	-	21.1 ± 19.1
**Liu *et al.*^18^, China**	rHR	39	50.0	37/2	2.09 ± 0.68	33.4	37 (94.9)	24
	Ablation	41	48.9	39/2	1.82 ± 0.82	21.9	39 (95.1)	24
**Lu *et al.*^31^, China**	rHR	138	50.1	112/26	2.8 ± 1.9	>2 years (84)	96 (69.6)	37.6
	Ablation	194	52.9	162/32	1.9 ± 0.9	>2 years (67)	134 (69.1)	41.6
**Peng *et al.*^32^, China**	rHR	79	55	59/20	≤3 (48)	≤1 year (46)	-	53.2 (4–96)
	Ablation	107	57	75/32	≤3 (73)	≤1 year (57)	-	52.3 (3–96)
**Ren *et al.*^33^, China**	rHR	145	51	127/18	2.0	≤2 years (71)	-	23 (3–88)
	Ablation	68	52	52/16	2.0	≤2 years (37)	-	23 (3–88)
**Saito *et al.*^34^, Japan**	rHR	17	-	-	-	-	-	-
	Ablation	26	-	-	-	-	-	-
**Song *et al.*^35^, Korea**	rHR	39	52.5	32/7	2.2 ± 1.1	20.9	23 (59)	36.3 (0.8–126.6)
	Ablation	178	55.4	156/22	1.7 ± 0.6	18.0	130 (73.0)	44.7 (5.6–139.8)
**Sun *et al.*^36^, Taiwan**	rHR	43	60	-	1.9 (0.8–3.0)	26	36 (83.7)	53
	Ablation	57	63	-	1.8 (1.0–3.0)	14	50 (87.7)	54
**Umeda *et al.*^37^, Japan**	rHR	29	≥65 (16)	-	3.2 ± 0.57	21.2	-	48
	Ablation	58	≥65 (37)	-	2.1 ± 0.3	21.2	-	48
**Wang *et al.*^38^, China**	rHR	128	50.2	89/39	2.4 ± 0.9	15.1	66 (51.6)	-
	Ablation	162	52.7	107/55	2.3 ± 0.7	14.1	-	-
**Xia *et al.*^19^, China**	rHR	120	52.4	96/24	2.9 (1.0–5)	29.5	50 (41.7)	44.3 (4.3–90.6)
	Ablation	120	53.5	94/26	2.7 (1.0–4.8)	26.3	55 (45.8)	44.3 (4.3–90.6)
**Xiao *et al.*^39^, China**	rHR	11	≤60 (8)	5/6	≤5 (8)	≥1 year (8)	-	-
	Ablation	24	≤60 (19)	11/13	≤5 (23)	≥1 year (23)	-	-
**Yan *et al.*^40^, China**	rHR	34	67.7	25/9	3.8 ± 0.7	11.7	14 (41.1)	-
	Ablation	22	68.4	15/7	3.9 ± 0.6	11.4	11 (50)	-
**Yin *et al.*^41^, China**	rHR	57	57	52/5	3.2	29	39 (68.4)	35 (6–60)
	Ablation	51	60	48/3	2.6	24	30 (58.8)	37 (7–60)
**Zhang *et al.*^42^, China**	rHR	69	-	-	3.5	14	61 (88.4)	-
	Ablation	99	-	-	2.1	15	76 (76.8)	-
**Zhang *et al.*^43^, China**	rHR	27	47	25/2	3.2 ± 1.1	36	-	32 (9–118)
	Ablation	39	52	37/2	2.7 ± 1.1	30	-	28 (2–79)
**Zhong *et al.*^44^, China**	rHR	307	53.2	229/78	≥3 (172)	≤1 year (80)	180 (58.6)	54 (1–178)
	Ablation	540	53.6	408/132	≥3 (115)	≤1 year (253)	304 (56.3)	49.3 (1–156)
**Chua *et al.*^45^, Singapore**	rHR	92	60	87/5	3.0 (2.2–4.0)	28.0	48 (52.2)	-
	Ablation	127	63	92/35	3.2 (2.2–4.5)	11.1	88 (69.3)	-
**Wei *et al.*^46^, China**	rHR	80	>45y (60)	69/11	≥3 (9)	≤1 year (25)	-	31 (7–63)
	Ablation	46	>45y (37)	33/13	≥3 (3)	≤1 year (28)	-	31 (7–63)
**Matsumoto *et al.*^47^, Japan**	rHR	23	66	19/4	3.2 (0.9–10.5)	-	16 (69.6)	43.2 (1.2–150)
	Ablation	11	67	8/3	2.0 (1.5–9.6)	-	6 (54.5)	-

* Mean or median. -, not reported; F, female; M, male; rHR, repeat hepatic resection.

### Perioperative morbidity and mortality

Perioperative morbidity rates were reported in 18 studies^18–20,22–24,27,30–33,35,36,40,42,44–46^. Median morbidity rate was higher in the rHR group (17.0 per cent, range 5.5–88.2 per cent) than in the ablation group (3.3 per cent, range 0–36.3 per cent). Common morbidities in the rHR group included hepatic insufficiency, pleural effusion, ascites, and biliary fistula, whereas bile leakage and abdominal haemorrhage were the most frequent morbidities in the ablation group. Perioperative mortality rates were reported in 21 studies^18–24,26,27,30–33,35,36,40–44^, and no significant differences were observed in the median mortality rate between the rHR group (0 per cent, range 0–2.9 per cent) and the ablation group (0 per cent, range 0–2.1 per cent) (*Table S3*).

### OS and RFS

**Fig. 2:**
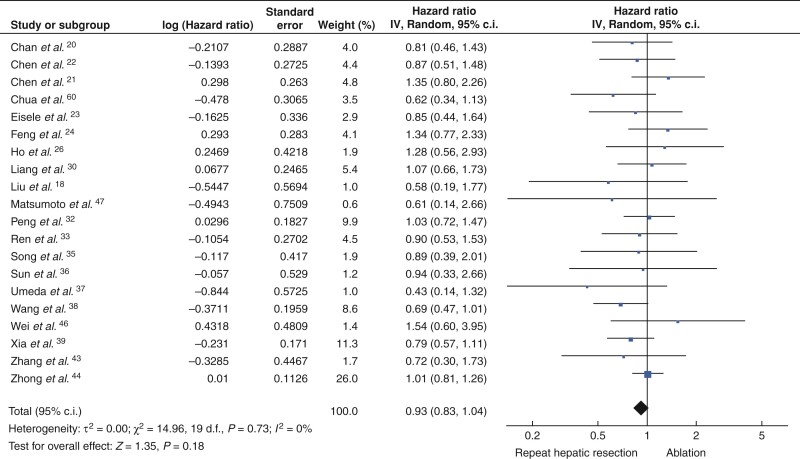
Forest plot comparing overall survival after repeat hepatic resection or ablation

**Fig. 3:**
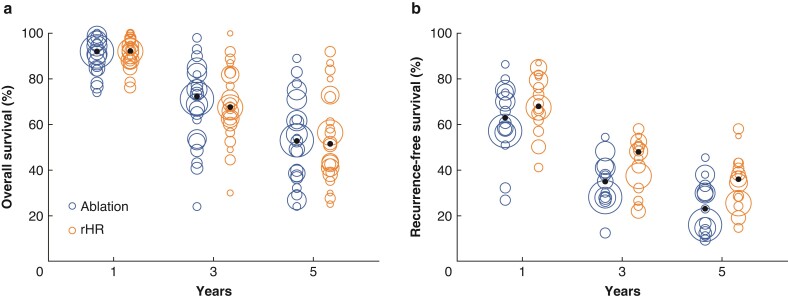
Bubble plots of 1-, 3-, and 5-year survival of patients with recurrent hepatocellular carcinoma after repeat hepatic resection or ablation

**Fig. 4:**
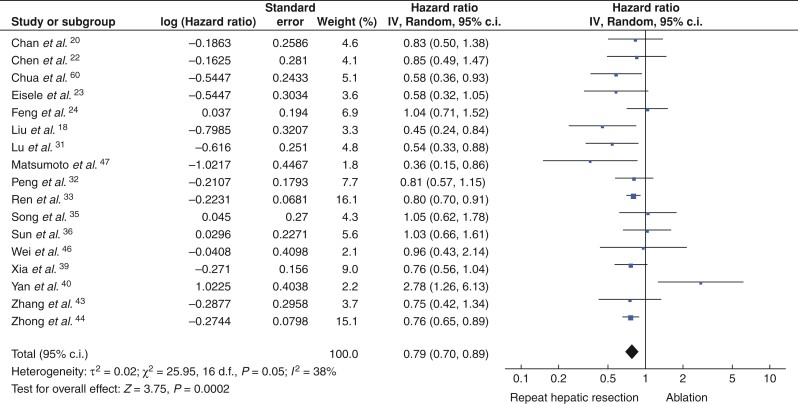
Forest plot comparing recurrence-free survival after repeat hepatic resection or ablation

HRs of OS was extracted from 20 studies. Patients in the rHR and ablation groups had similar OS (HRs 0.93, 95 per cent c.i. 0.83 to 1.04, *P* = 0.18) (*Fig. 2*) and median OS rates at 1 year (92.3 per cent *versus* 92.1 per cent), 3 years (67.7 per cent *versus* 72.3 per cent), and 5 years (51.5 per cent *versus* 52.9 per cent; *Fig. 3a*). HRs of RFS was extracted from 17 studies. Patients in the rHR group had significantly higher RFS (HRs 0.79, 95 per cent c.i. 0.70 to 0.89, *P* < 0.001) (*Fig. 4*) as well as higher median RFS rates at 1 year (68.3 per cent *versus* 63.3 per cent), 3 years (48.1 per cent *versus* 35.2 per cent), and 5 years (36.2 per cent *versus* 23.0 per cent) (*Fig. 3b*).

Meta-analysis was conducted using a random-effects model. HRs are shown with 95 per cent confidence intervals (95 per cent c.i.). *P* = 0.25.

**a** Overall survival. **b** Recurrence-free survival. The size of each bubble represents the sample size of the given study

Meta-analysis was conducted using a random-effects model. HRs are shown with 95 per cent confidence intervals (95 per cent c.i.). *P* < 0.001.

### Sensitivity analysis and publication bias

Sensitivity analysis showed that excluding any one of the studies, including the one reporting microwave ablation^43^, did not significantly affect the pooled results (*Figs. S1* and *S2*). Similar results were obtained when all studies were meta-analysed with a random- or fixed-effect model. However, visual inspection of funnel plots suggested the possibility of publication bias (*Figs. S3* and *S4*).

## Discussion

Postoperative tumour recurrence is the most important factor affecting the long-term survival of patients with HCC after hepatic resection. Previous studies have shown that rHR and ablation are the most effective methods for treating recurrent HCC^8,13,48^ although the 5-year re-recurrence rate remains high. In the present meta-analysis, safety, and efficacy of rHR and ablation were compared using a larger sample than in previous studies^8–13^. Both therapeutic approaches provided similar OS, but rHR was associated with longer RFS at the expense of higher perioperative morbidity.

Earlier meta-analyses involving studies with small samples indicated that radiofrequency and microwave ablation have similar efficacy for primary untreated HCC^49,50^, suggesting that these two percutaneous techniques could be aggregated in the present analysis. Of the 30 studies selected, only 1^43^ compared the efficacy of microwave ablation and rHR reporting similar OS, but slightly higher RFS for rHR.

The present results are consistent with the findings of previous meta-analyses^51–53^, but show higher median 5-year OS (>50 per cent) after both treatments than previously reported (35.2 per cent and 48.3 per cent for rHR and for ablation respectively)^8^. Four small meta-analyses concluded that rHR was associated with better OS than ablation^10–13^, whereas another study reported similar RFS for the two treatments^9^. This discrepancy may be explained by the smaller sample size of previous studies.

A recent meta-analysis of 7 RCTs and 18 matched non-RCTs concluded that hepatic resection and radiofrequency ablation were associated with similar OS for patients with primary untreated HCC satisfying the Milan criteria, but that hepatic resection may be associated with better RFS and lower rate of local recurrence^1^. Consistent with these results, in this meta-analysis both treatments achieved similar 1-, 3-, and 5-year OS in patients with recurrent HCC, whereas rHR was associated with considerably higher RFS. In previous studies, 14.9 per cent of patients with HCC showed insufficient margins^54^ and shorter time to recurrence after ablation^19,44^. The significant difference in RFS between the two treatment groups in the present meta-analysis might be explained by incomplete ablation. In contrast, the similar OS values might reflect the fact that some patients received one or more additional treatments after tumour recurrence or re-recurrence^44^, that led to improved OS.

Although radiofrequency ablation is commonly used to treat HCC with tumour diameter more than 3 cm, it is currently considered best for HCC tumours less than 3 cm^55^. Radiofrequency ablation removes HCC with diameters of 3–5 cm much less effectively than in smaller tumours, translating to greater risk of local recurrence^56^. The efficacy of radiofrequency ablation also decreases gradually with increasing tumour number and diameter^57^. These findings suggest that tumour diameter should be considered when selecting treatment options for recurrent HCC. Unfortunately, subgroup analyses based on tumour diameter or number was not possible in the present meta-analysis, as most of the included studies reported only tumour stage.

The present results should be interpreted carefully considering several limitations. Most of the included studies were observational, indicating that additional well designed RCTs should be conducted in the future. Moreover, rHR and ablation may have different indications for recurrent HCC depending on tumour diameter, location, and patient characteristics. As most of the studies reported only data for recurrent HCC within Milan criteria, these results may not be generalizable to other patients. Patients in the included studies may have received one or more additional treatments after rHR or ablation, which may have affected their prognosis. For instance, several tyrosine kinase and immune-checkpoint inhibitors have recently been identified as first- or second-line therapy for patients with advanced or unresectable HCC^58–61^. Thus, the combination of rHR or local ablation with such inhibitors may improve the survival of patients with recurrent HCC. Finally, potential publication bias was observed in the funnel plots. Future meta-analysis with larger sample size may change the findings of the present study.

Despite these limitations, this meta-analysis provides evidence that rHR and local ablation are associated with similar OS in patients with recurrent HCC. rHR seems to be associated with better RFS, whereas local ablation leads to lower perioperative morbidity. These nuances highlight the need for individualized, multidisciplinary strategies when treating recurrent HCC.

## Supplementary Material

zrac036_Supplementary_Data

## Data Availability

All supporting data are included within the main article and its supplementary files.
