# Supplementary material for: Repeat hepatic resection versus percutaneous ablation for the treatment of recurrent hepatocellular carcinoma: meta-analysis
Source: BJS Open. 2022 Apr 28;6(2):zrac036. doi: 10.1093/bjsopen/zrac036 (PMC9048940; doi:10.1093/bjsopen/zrac036)
Supplement: zrac036_Supplementary_Data [file zrac036_supplementary_data.zip › Supplementary_Figures.docx]

**
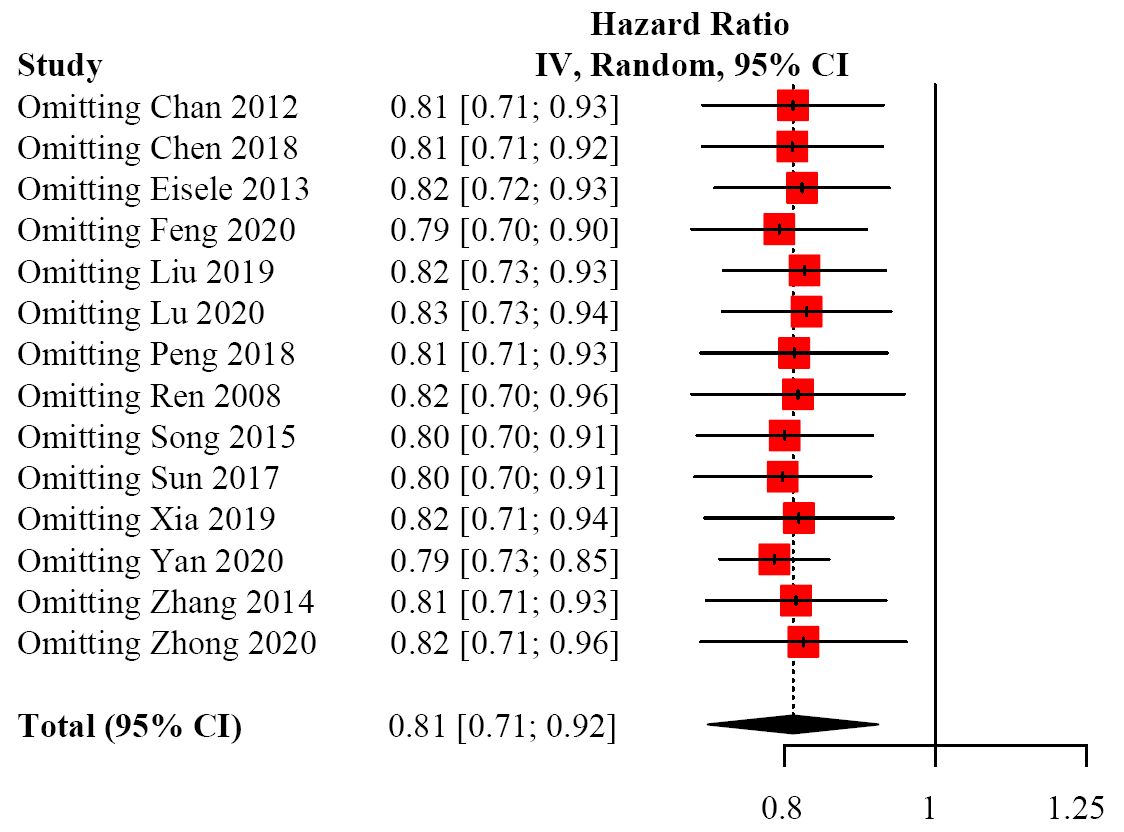
**

**Figure S1.** Sensitivity analysis for recurrence-free survival by excluding any one study.


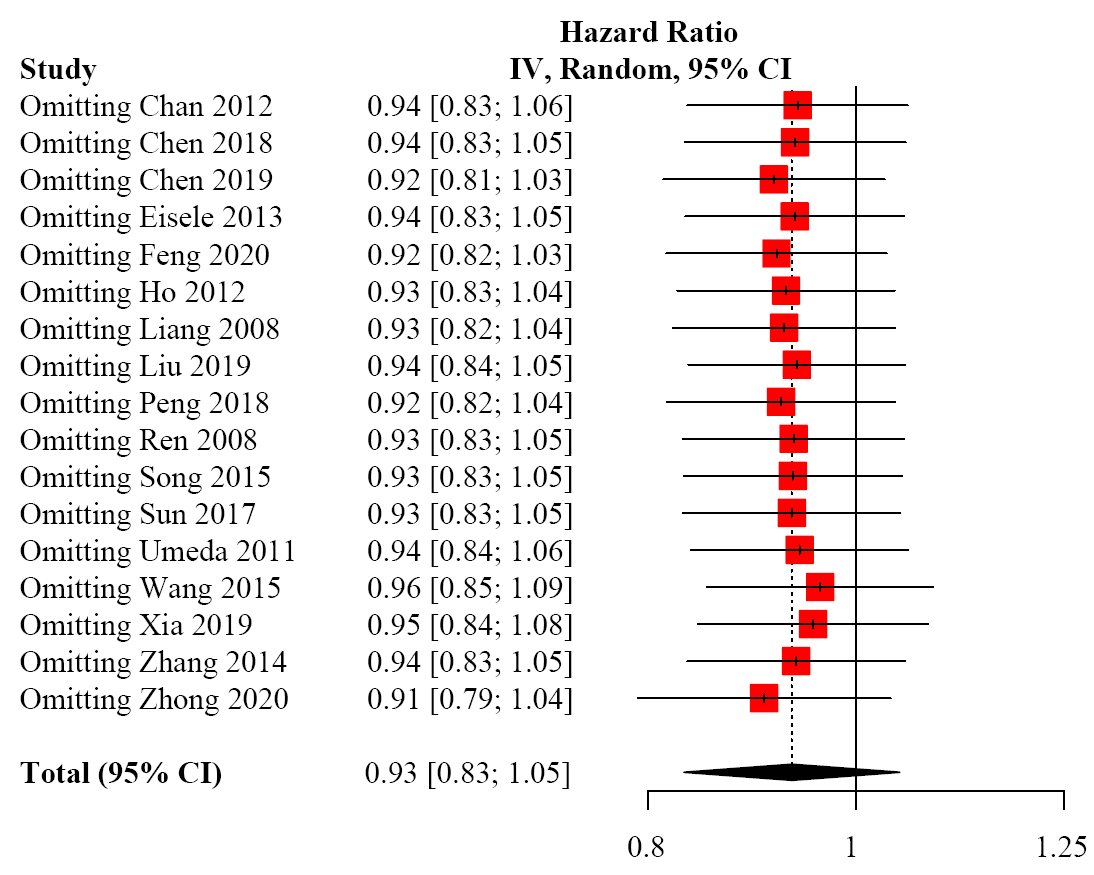
**Figure S2.** Sensitivity analysis for overall survival by excluding any one study.


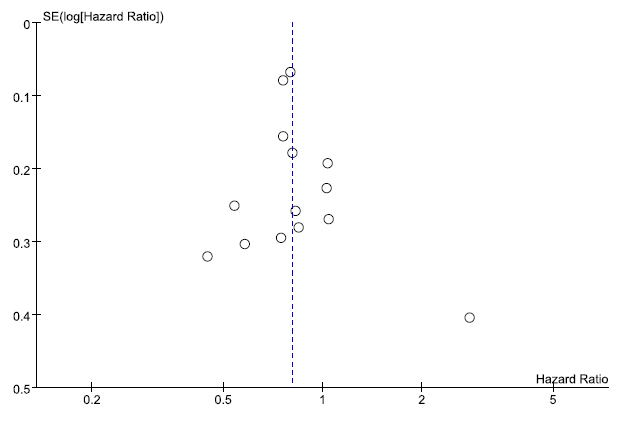
**Figure S3.** Funnel plot of recurrence-free survival among patients after repeat hepatic resection or ablation.


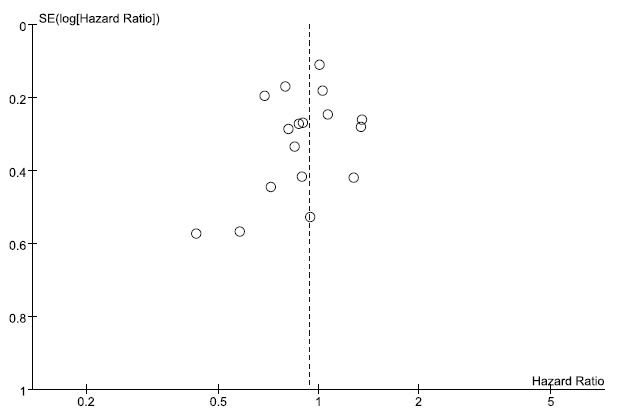
**Figure S4.** Funnel plot of overall survival among patients after repeat hepatic resection or ablation.
