# Supplementary material for: Repeat hepatic resection versus percutaneous ablation for the treatment of recurrent hepatocellular carcinoma: meta-analysis
Source: BJS Open. 2022 Apr 28;6(2):zrac036. doi: 10.1093/bjsopen/zrac036 (PMC9048940; doi:10.1093/bjsopen/zrac036)
Supplement: zrac036_Supplementary_Data [file zrac036_supplementary_data.zip › Supplementary_Table_1.docx]

Table S1. Quality assessment of the included randomized controlled trials according to the Cochrane Intervention Systematic Review Manual.

| Study | Sequence generation of randomization | Allocation concealment | Blinding of patients | Blinding of personnel | Blinding of outcome assessment | Study quality |
| --- | --- | --- | --- | --- | --- | --- |
| Xia 2019 | Yes | Yes | No | No | No | Moderate |
| Liu 2019 | Yes | Yes | No | No | No | Moderate |
