# Supplementary material for: Repeat hepatic resection versus percutaneous ablation for the treatment of recurrent hepatocellular carcinoma: meta-analysis
Source: BJS Open. 2022 Apr 28;6(2):zrac036. doi: 10.1093/bjsopen/zrac036 (PMC9048940; doi:10.1093/bjsopen/zrac036)
Supplement: zrac036_Supplementary_Data [file zrac036_supplementary_data.zip › Supplementary_Table_2.docx]

Table S2. Quality assessment of the included non-randomized controlled trials based on the Newcastle-Ottawa Scale.

| Study | Selection stars | Comparability stars | Outcome stars | Total stars | Study quality |
| --- | --- | --- | --- | --- | --- |
| Chan 2012 | 3 | 1 | 3 | 7 | High |
| Chen 2018 | 3 | 2 | 2 | 7 | High |
| Chen 2019 | 3 | 2 | 2 | 7 | High |
| Eisele 2013 | 3 | 0 | 2 | 5 | Moderate |
| Feng 2020 | 3 | 2 | 2 | 7 | High |
| Hirokawa 2011 | 3 | 2 | 3 | 8 | High |
| Ho 2012 | 3 | 1 | 2 | 6 | High |
| Huang 2013 | 3 | 1 | 2 | 6 | High |
| Kawano 2009 | 3 | 1 | 3 | 7 | High |
| Kim 2020 | 3 | 1 | 2 | 6 | High |
| Liang 2008 | 3 | 2 | 1 | 6 | High |
| Lu 2020 | 3 | 1 | 1 | 5 | Moderate |
| Peng 2018 | 3 | 2 | 2 | 7 | High |
| Ren 2008 | 3 | 2 | 3 | 8 | High |
| Saito 2020 | 2 | 1 | 2 | 5 | Moderate |
| Song 2015 | 3 | 2 | 2 | 7 | High |
| Sun 2017 | 3 | 2 | 3 | 8 | High |
| Umeda 2011 | 2 | 1 | 3 | 6 | High |
| Wang 2015 | 3 | 2 | 3 | 8 | High |
| Xiao 2019 | 3 | 2 | 3 | 8 | High |
| Yan 2020 | 3 | 2 | 3 | 8 | High |
| Yin 2019 | 3 | 2 | 3 | 8 | High |
| Zhang 2013 | 3 | 1 | 2 | 6 | High |
| Zhang 2014 | 3 | 2 | 1 | 6 | High |
| Zhong 2021 | 3 | 2 | 3 | 8 | High |
| Chua 2021 | 3 | 2 | 3 | 8 | High |
| Wei 2021 | 3 | 1 | 2 | 6 | High |
| Matsumoto 2021 | 3 | 2 | 3 | 8 | High |
