# Supplementary material for: Repeat hepatic resection versus percutaneous ablation for the treatment of recurrent hepatocellular carcinoma: meta-analysis
Source: BJS Open. 2022 Apr 28;6(2):zrac036. doi: 10.1093/bjsopen/zrac036 (PMC9048940; doi:10.1093/bjsopen/zrac036)
Supplement: zrac036_Supplementary_Data [file zrac036_supplementary_data.zip › Supplementary_Table_3.docx]

Table S3. Outcomes of included studies.

| **Study** | **Treatment** | **Sample size** | **Mortality, %** | **Morbidity, %** | **RFS, %** | | | **OS, %** | | | **Hazard ratio  (95% CI), OS** | **Hazard ratio (95% CI), RFS** |
| --- | --- | --- | --- | --- | --- | --- | --- | --- | --- | --- | --- | --- |
|  |  |  |  |  | 1-yr | 3-yr | 5-yr | 1-yr | 3-yr | 5-yr |  |  |
| Chan 2012 | rHR | 29 | 0 | 24.1 | 41.1 | 24.2 | 24.2 | 89.7 | 56.5 | 35.2 | 0.81 (0.46-1.44) | 0.83 (0.50-1.39) |
|  | Ablation | 45 | 2.2 | 2.2 | 32.2 | 12.4 | 9.3 | 83.7 | 43.1 | 29.1 |  |  |
| Chen 2018 | rHR | 48 | 2.1 | 25 | 73.1 | 49.7 | 40.7 | 76.3 | 52.5 | 42.6 | 0.87 (0.51-1.48) | 0.85 (0.49-1.46) |
|  | Ablation | 57 | 0 | 0 | 69.5 | 37.8 | 33.1 | 78.2 | 40.8 | 36.7 |  |  |
| Chen 2019 | rHR | 77 | 1.3 | - | - | - | - | 88.8 | 68.8 | 51.1 | 1.35 (0.80-2.26) | - |
|  | Ablation | 82 | 0 | - | - | - | - | 91.4 | 73.4 | 61.1 |  |  |
| Eisele 2013 | rHR | 27 | 0 | 14.8 | 82 | 45 | 28 | 100 | 68 | 39 | 0.85 (0.44-1.67) | 0.58 (0.32-1.05) |
|  | Ablation | 27 | 0 | 3.7 | 51 | 30 | 11 | 96 | 62 | 32 |  |  |
| Feng 2020 | rHR | 91 | 0 | 9.1 | 50.2 | 21.9 | 19.2 | 87.7 | 62.9 | 38.1 | 1.34 (0.77-2.34) | 1.038 (0.71-1.52) |
|  | Ablation | 199 | 0 | 0.5 | 56.5 | 27.9 | 14.6 | 90.7 | 69.0 | 55.6 |  |  |
| Hirokawa 2011 | rHR | 10 | - | - | 87.0 | 55.0 | 55.0 | 100 | 100 | 80.0 | - | - |
|  | Ablation | 21 | - | - | 74.0 | 38.0 | 38.0 | 100 | 82.0 | 54.0 |  |  |
| Ho 2012 | rHR | 54 | 0 | - | - | - | - | 98.0 | 83.0 | 72.0 | 1.28 (0.56-2.89) | - |
|  | Ablation | 50 | 0 | - | - | - | - | 95.0 | 90.0 | 83.0 |  |  |
| Huang 2013 | rHR | 66 | 0 | 18.1 | 43.9 | 14.4 | 8.2 | 89.5 | 54.3 | 28.8 | - | - |
|  | Ablation | 46 | 0 | 8.7 | 56.9 | 12.4 | 5.0 | 82.6 | 50.8 | 20.5 |  |  |
| Kawano 2009 | rHR | 13 | - | - | - | - | - | 100 | 49.0 | 25.0 | - | - |
|  | Ablation | 33 | - | - | - | - | - | 100 | 93.0 | 78.1 |  |  |
| Kim 2020 | rHR | 45 | - | - | 76.2 | 58.1 | 58.1 | 97.1 | 92.0 | 92.0 | - | - |
|  | Ablation | 171 | - | - | 75.1 | 48.3 | 38.0 | 97.0 | 85.0 | 75.0 |  |  |
| Liang 2008 | rHR | 44 | 0 | 68.2 | - | - | - | 78.6 | 44.5 | 27.6 | 1.07 (0.66-1.72) | - |
|  | Ablation | 66 | 0 | 3.0 | - | - | - | 76.6 | 48.6 | 39.9 |  |  |
| Liu 2019 | rHR | 39 | 0 | 30.7 | 69.2 | - | - | 92.3 | - | - | 0.58 (0.19-1.81) | 0.45 (0.24-0.82) |
|  | Ablation | 41 | 0 | 7.3 | 26.8 | - | - | 85.4 | - | - |  |  |
| Lu 2020 | rHR | 138 | 0 | 7.2 | - | - | - | 91.8 | 82.0 | 72.9 | - | 0.54 (0.33-0.88) |
|  | Ablation | 194 | 0 | 2.6 | - | - | - | 94.4 | 75.4 | 61.7 |  |  |
| Peng 2018 | rHR | 79 | 1.3 | 17.7 | 64.8 | 41.6 | 38.3 | 84.8 | 60.2 | 51.9 | 1.03 (0.72-1.47) | 0.81 (0.57-1.17) |
|  | Ablation | 107 | 0 | 4.7 | 58.2 | 35.2 | 29.6 | 84.6 | 66.9 | 49.1 |  |  |
| Ren 2008 | rHR | 145 | 0 | 5.5 | 79.4 | 48.1 | 34.4 | 88.1 | 62.6 | 41.0 | 0.90 (0.53-1.52) | 0.80 (0.70-0.92) |
|  | Ablation | 68 | 0 | 1.5 | 58.0 | 27.8 | 12.4 | 94.7 | 65.1 | 37.3 |  |  |
| Saito 2020 | rHR | 17 | - | - | 81.0 | 52.0 | 38.0 | 95.0 | 87.0 | 87.0 | - | - |
|  | Ablation | 26 | - | - | 80.0 | 38.0 | 23.0 | 100 | 98.0 | 89.0 |  |  |
| Song 2015 | rHR | 39 | 2.6 | 7.7 | 66.1 | 48.5 | 43.1 | 88.8 | 88.8 | 83.9 | 0.89 (0.39-2.02) | 1.05 (0.62-1.78) |
|  | Ablation | 178 | 0 | 2.2 | 70.1 | 40.8 | 30.0 | 98.9 | 82.5 | 71.0 |  |  |
| Sun 2017 | rHR | 43 | 2 | 16 | 57.0 | 32.1 | 28.6 | 97.6 | 82.7 | 56.4 | 0.95 (0.3-2.67) | 1.03 (0.66-1.62) |
|  | Ablation | 57 | 0 | 7 | 60.8 | 26.6 | 16.6 | 98.2 | 77.2 | 52.6 |  |  |
| Umeda 2011 | rHR | 29 | - | - | - | - | - | 93.1 | 66.8 | 58.1 | 0.43 (0.14-1.22) | - |
|  | Ablation | 58 | - | - | - | - | - | 94.7 | 75.1 | 48.3 |  |  |
| Wang 2015 | rHR | 128 | - | - | - | - | - | 94.5 | 71.5 | 43.0 | 0.69 (0.47-1.06) | - |
|  | Ablation | 162 | - | - | - | - | - | 90.4 | 53.7 | 26.7 |  |  |
| Xia 2020 | rHR | 120 | 0 | 22.4 | 85.0 | 52.4 | 36.2 | 92.5 | 65.8 | 43.6 | 0.79 (0.57-1.10) | 0.76 (0.56-1.04) |
|  | Ablation | 120 | 0 | 7.3 | 74.2 | 41.7 | 30.2 | 87.5 | 52.5 | 38.5 |  |  |
| Xiao 2019 | rHR | 11 | - | - | - | - | - | 90 | 30 | 30 | - | - |
|  | Ablation | 24 | - | - | - | - | - | 74 | 24 | 24 |  |  |
| Yan 2020 | rHR | 34 | 2.9 | 88.2 | 61.8 | 26.5 | 14.7 | - | - | - | - | 2.78 (1.26-6.13) |
|  | Ablation | 22 | 0 | 36.3 | 86.4 | 54.5 | 45.5 | - | - | - |  |  |
| Yin 2019 | rHR | 57 | 0 | - | 68.4 | 39.4 | 26.6 | 78.9 | 50.5 | 29.7 | - | - |
|  | Ablation | 51 | 0 | - | 62.8 | 32.8 | 20.4 | 80.3 | 50.9 | 26.0 |  |  |
| Zhang 2013 | rHR | 69 | 1.4 | 15.9 | - | - | - | 68.2 | 45.5 | - | - | - |
|  | Ablation | 99 | 0 | 0 | - | - | - | 73.7 | 53.6 | - |  |  |
| Zhang 2014 | rHR | 27 | 0 | - | 66.7 | 50.7 | 43.4 | 96.2 | 76.9 | 61.2 | 0.72 (0.30-1.72) | 0.75 (0.42-1.33) |
|  | Ablation | 39 | 0 | - | 65.8 | 28.0 | 14.0 | 86.2 | 73.3 | 62.2 |  |  |
| Zhong 2021 | rHR | 307 | 0.6 | 59.6 | 67.4 | 37.5 | 25.5 | 92.1 | 67.4 | 56.4 | 1.01 (0.81-1.26) | 0.76 (0.65-0.89) |
|  | Ablation | 540 | 0.5 | 7.4 | 57.3 | 28.1 | 16.0 | 92.1 | 71.3 | 53.1 |  |  |
| Chua 2021 | rHR | 92 | 6.6 | 38.4 | 64.0 | 45.0 | 37.0 | 90.1 | 72.5 | 61.4 | 0.62 (0.34-1.12) | 0.58 (0.36-0.93) |
|  | Ablation | 127 | 1.6 | 11.8 | 45.0 | 25.0 | 19.0 | 94.2 | 62.6 | 36.9 |  |  |
| Wei 2021 | rHR | 80 | - | ≥ 3, 3.8 | 71.4 | 42.9 | 32.3 | 88.6 | 73.8 | 59.0 | 1.54 (0.60-3.97) | 0.96 (0.43-2.14) |
|  | Ablation | 46 | - | ≥ 3, 2.2 | 68.6 | 51.0 | 34.0 | 94.3 | 82.9 | 71.4 |  |  |
| Matsumoto 2021 | rHR | 23 | - | - | 69.6 | 50.6 | 40.5 | 100 | 100 | 89.3 | 0.61 (0.14-2.77) | 0.36 (0.15-0.87) |
|  | Ablation | 11 | - | - | 36.4 | 18.2 | 18.2 | 100 | 90.9 | 90.9 |  |  |

CI, confidence interval; OS, overall survival; RFS, recurrence-free survival; rHR, repeat hepatic resection.
